# Using routine referral data for patients with knee and hip pain to improve access to specialist care

**DOI:** 10.1186/s12891-020-3087-x

**Published:** 2020-02-03

**Authors:** Kate Button, Irena Spasić, Rebecca Playle, David Owen, Mandy Lau, Liam Hannaway, Stephen Jones

**Affiliations:** 1 School of Healthcare Sciences, Cardiff University, Eastgate House, Newport Road, Cardiff, CF24 0AB UK; 2 Physiotherapy Department, Cardiff and Vale University Health Board, Cardiff, UK; 3 School of Computer Science & Informatics, Cardiff University, Cardiff, UK; 4 Centre for Trials Research, Cardiff University, Cardiff, UK; 5 Brynderwen Surgery, St Mellons, Cardiff, UK; 6 Trauma and Orthopaedics, Cardiff and Vale Orthopaedic Centre, University Hospital Llandough, Cardiff and Vale UHB, Cardiff, UK

**Keywords:** Knee, Hip, Musculoskeletal, Care pathway, Text mining

## Abstract

**Background:**

Referral letters from primary care contain a large amount of information that could be used to improve the appropriateness of the referral pathway for individuals seeking specialist opinion for knee or hip pain. The primary aim of this study was to evaluate the content of the referral letters to identify information that can independently predict an optimal care pathway.

**Methods:**

Using a prospective longitudinal design, a convenience sample of patients with hip or knee pain were recruited from orthopaedic, specialist general practice and advanced physiotherapy practitioner clinics. Individuals completed a Knee or hip Osteoarthritis Outcome Score at initial consultation and after 6 months. Participant demographics, body mass index, medication and co-morbidity data were extracted from the referral letters. Free text of the referral letters was mapped automatically onto the Unified Medical Language System to identify relevant clinical variables. Treatment outcomes were extracted from the consultation letters. Each outcome was classified as being an optimal or sub-optimal pathway, where an optimal pathway was defined as the one that results in the right treatment at the right time. Logistic regression was used to identify variables that were independently associated with an optimal pathway.

**Results:**

A total of 643 participants were recruited, 419 (66.7%) were classified as having an optimal pathway. Variables independently associated with having an optimal care pathway were lower body mass index (OR 1.0, 95% CI 0.9 to 1.0 *p* = 0.004), named disease or syndromes (OR 1.8, 95% CI 1.1 to 2.8, *p* = 0.02) and taking pharmacologic substances (OR 1.8, 95% CI 1.0 to 3.3, *p* = 0.02). Having a single diagnostic procedure was associated with a suboptimal pathway (OR 0.5, 95% CI 0.3 to 0.9 *p* < 0.001). Neither Knee nor Hip Osteoarthritis Outcome scores were associated with an optimal pathway. Body mass index was found to be a good predictor of patient rated function (coefficient − 0.8, 95% CI -1.1, − 0.4 *p* < 0.001).

**Conclusion:**

Over 30% of patients followed sub-optimal care pathway, which represents potential inefficiency and wasted healthcare resource. A core data set including body mass index should be considered as this was a predictor of optimal care and patient rated pain and function.

## Background

The current recommended pathway for long term conditions, including adults with knee and hip pain, is management in primary care and referral to a multi-professional assessment and treatment clinic if specialist opinion is required [1–3]. However, there are a number of variations in the current care pathway, which represent potential inefficiency in resource use and standards of care [4] and importantly delay for patients [5]. This includes variation in where clinics are based, what profession assesses and treats patients and in the care given [5–7]. With an aging population and rising treatment expectations the burden on healthcare resources is increasing [8]. Therefore, triage methods that streamline patients to maximise efficiency and ensure individuals receive optimal care for their needs are required. This includes ensuring early access to non-surgical treatment options such as physiotherapy, pain medication and dieticians, that treatment is given in a timely manner and in a suitable setting to meet patient needs [1, 9]. For example, an optimal outcome from a surgical consultation based on individual circumstances would be a referral for surgery, whereas sub-optimal outcome would be no definitive treatment.

To try and improve efficiency and resource use in secondary care, referral prioritisation systems have been developed for hip and or knee pain and tested to fast-track cases for surgical opinion based on general practitioner referral information [10, 11]. The limitation of these systems is that the prioritisation criteria has lacked sensitivity and specificity as individuals move between surgical and conservative pathways. The quality of the research means that insufficient evidence exists regarding which predictor variables can be used to inform decision making. Furthermore, the criterion for prioritisation rely on patient rated outcome measures and x-ray, but for conditions such as knee osteoarthritis it is recommended that decision to refer for a surgical opinion is based on discussion between patients and clinician [1]. Referral letters often contain information that underpins referral decision making, e.g. narrative description of the ways in which a given joint condition is impacting the patient’s everyday activities. However, such information has never been explored systematically in research on treatment prioritisation/ streamlining systems for orthopaedic conditions.

Analysis of free text data in general practitioner referral letters can be done using text mining techniques to create variables that can be used alongside demographic and health related data and has the potential to improve treatment prioritisation. This technique has been used successfully in the evaluation of radiology reports and health correspondence on web-based health communities and questionnaires [12–15].

Management of these routine data from general practitioner referral letters may provide invaluable information that can predict where and by whom an individual is best seen by identifying associations between the referral information and treatment outcome [13]. Therefore, it could be used for early streamlining of the type of care an individual should receive and better resource allocation within the referral pathway. This adds to the findings of previously reported knee and or hip specific prioritisation and streamlining systems that have not included this data [8, 9].

The primary aim of the study was to identify factors from the general practitioner referral letters that can predict who would receive an optimal versus sub-optimal care pathway at the time of consultation with a specialist in an advanced physiotherapy practice, specialist GP or orthopaedic clinic. The secondary aims were to:

- Identify factors from the GP referral letters that can predict patient rated pain and function at time of consultation with a specialist and after 6 months.
- Describe the characteristics of the care pathway for hip and / or knee pain according to specialist clinic type.

These findings could be used to streamline the referral process and provide recommendations in pathway re-design and streamlining patients to optimise care.

## Methods

**Fig. 1:**
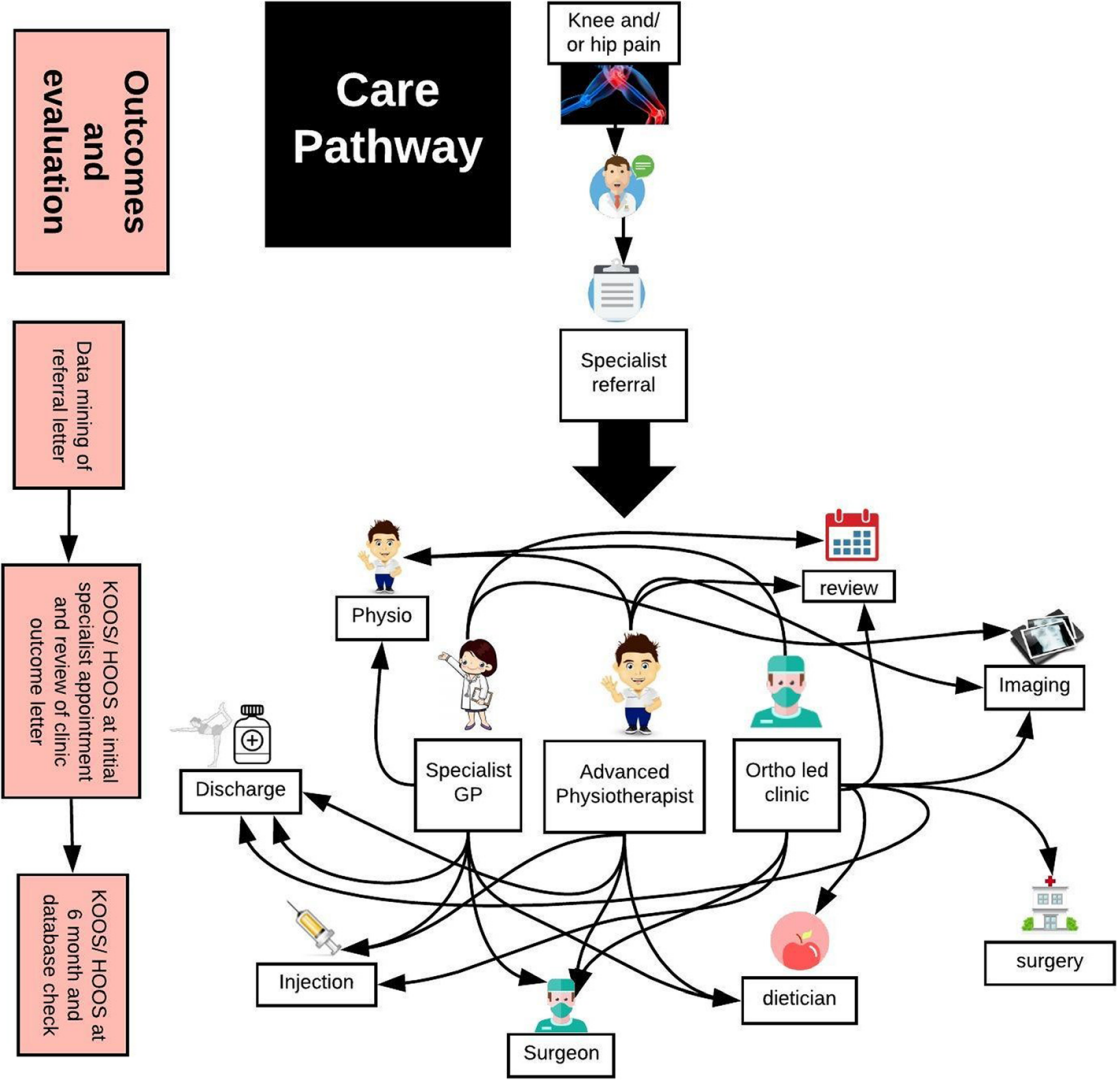
Care pathway for hip and knee pain

The research design was a prospective longitudinal design. All data were collected between August 2016 to January 2017 and follow-up data collection was completed in June 2018. All participants were recruited from the musculoskeletal service at one University Health Board, an administrative unit within the National Health Service. The care pathway is illustrated in Fig. 1. A consecutive sample of patients with hip and or knee pain that had been referred by their general practice for specialist opinion were screened for inclusion from orthopedic surgeon led orthopaedic clinics, specialist general practitioner clinics (specialist GP) and advanced physiotherapy practitioner clinics. The aim of the clinics was to give a specialist opinion and have a definitive treatment outcome. Individuals were eligible to take part in the study if they were referred by their general practitioner for knee and/or hip pain, if they were aged 18 or over, could provide informed consent and understand English sufficiently to be able to complete the questionnaires. The exclusion criteria were knee/hip pain secondary to other health conditions such as rheumatoid arthritis, pain secondary to knee/hip replacement, surgery for the same knee/hip within the past 12 months or already having received treatment at the primary/secondary care interface for the same condition within the previous 6 months.

A sample of 634 participants were recruited. The sample size was determined based on the volume of consecutive monthly referrals received by the service for the 6 month duration of the project. No sample size calculation was performed but we allowed for a minimum of 10 cases per potential predictor variable. This sample size also allowed for incomplete data such as missing referral letters or data on referrals. Based on 26 predictor outcomes that were explored for inclusion in the model this allowed for 23 subjects per variable on average.

At the time of attendance at their specialist consultation individuals were asked to complete a questionnaire in order to calculate knee or hip osteoarthritis outcome score (KOOS or HOOS). The KOOS and HOOS scores are validated and reliable patient rated outcome measures for assessing pain, symptoms, activities of daily living, sport and quality of life in individuals with knee and hip conditions [16].

Key data were extracted from their referral letter including age, gender, postcode, body mass index (BMI), medication, smoking status, co-morbidity data and free text referral information. From the co-morbidity data the Charlston co-morbidity index for each participant was calculated. A score of 0 indicates no co-morbidity and a score of 3 indicates high level of co-morbidity [17]. The outcome of the consultation such as the recommended treatment or further referral (e.g. consultation notes i.e. physiotherapy, surgery, discharge, follow-up, injection, imaging, dietician or orthopedic consultant referral) was extracted from the clinic outcome letter and checked by a second member of the research team.

Clinical narratives (free text) within the general practitioner referral letters were coded automatically in preparation for subsequent statistical analysis. The coding was performed against the MetaThesaurus included in the Unified Medical Language system [18]. The MetaThesaurus is a large biomedical thesaurus that is organised by concept (i.e. meaning) whose various names (or terms) are drawn from around 200 source vocabularies, e.g. SNOMED, HL7, ICD-10, DrugBank, MedDRA, etc. The free-text content of referral letters was mapped against the MetaThesaurus using MetaMap [19], a dictionary lookup application developed specifically to flexibly match biomedical terms against text and map their occurrences back to the corresponding concept identifier. We limited the lookup to concepts of certain types using the categorisation of the MetaThesaurus concepts into the Semantic Network, a taxonomy of over 130 semantic types. Specifically, we focused on the following semantic types: “Diagnostic Procedure”, “Daily or Recreational Activity”, “Disease or Syndrome”, “Finding”, “Functional Concept”, “Health Care Activity”, “Injury or Poisoning”, “Occupational Activity”, “Physiologic Function”, “Pharmacologic Substance”, “Sign or Symptom”, “Tissue”, “Therapeutic or Preventive Procedure”, “Body related concept” (an aggregation of the semantic types: “Body Location or Region”, “Body Part, Organ, or Organ Component”, “Body Space or Junction”). The MetaMap output was used to count the number of mentions per concept. This ensured that statistical analysis was based on the underlying meaning and not on surface textual representation. For example, synonyms such as ‘edema’ and ‘swelling’ would be represented by the same code. In addition, all concept mentions were also aggregated across semantic types. For example, both ‘edema’ and ‘pain’ would count toward a ‘Sign or Symptom’. Once extracted, all coded data were formatted and stored in a relational database to allow easy export for further statistical analysis.

At 6 months post the initial consultation, participants were contacted by post and asked to complete KOOS/HOOS questionnaires. The hospital database was checked to evaluate if the treatment they had been referred for had been completed, if they were still waiting or if an alternative treatment had been given.

The primary aim of this study was to identify factors predicting an optimal care pathway. An optimal care pathway was defined as one that minimises delayed treatment for the patient and results in care being delivered in a timely fashion in the right setting and by the right person. This definition was based on the literature [9, 20–22] and by steering committee consensus who included two surgeons, a general practitioner, a physiotherapist, a member of the public, two research assistants, two medical statisticians and a computer scientist. Using this definition, a grid of optimal/ sub-optimal treatment outcomes per clinic type was created by two clinician reviewers (advanced physiotherapy practitioner and orthopaedic surgeon) independently reviewing the treatment outcome types. Agreement was achieved by consensus with a third member of the research team who was not a healthcare professional. The grid was then reviewed by the steering committee. This was then applied to the outcome data by the statistician (Table 1). Examples of optimal care from an orthopaedic clinic would be listed for surgical procedure. Non-optimal care from an orthopaedic clinic would be referral to non-surgical treatments such as physiotherapy as this would have been expected to have been done before specialist opinion in a surgeon led clinic. Optimal care from advanced physiotherapy practitioner or specialist GP may be discharge or referral for non-surgical treatment. Sub-optimal care would be an outcome of further review with no definitive outcome [1]. In the given pathway, MRI imaging and injection were not routinely available in primary care and, therefore, were rated as optimal outcomes for the advanced physiotherapy practitioner and specialist GP clinic.

**Table 1:** Definitions of optimal and suboptimal treatment outcomes per clinic type

Combination of outcomes	Optimal/ suboptimal outcome combinations		
	Specialist GP clinic	Advanced physiotherapy practitioner clinic	Orthopaedic clinic
Single outcomes			
Consultant^a^	Sub-optimal^a^	sub-optimal^a^	sub-optimal^a^
Physio^b^	optimal^b^	optimal^b^	sub-optimal^b^
Dietician^b^	optimal^b^	optimal^b^	sub-optimal^b^
Monitoring^c^	sub-optimal^c^	sub-optimal^c^	sub-optimal^c^
Surgery^d^	sub-optimal^d^	sub-optimal^d^	optimal
Imaging^e^	optimal^e^	optimal^e^	sub-optimal^e^
Injection^e^	optimal^e^	optimal^e^	sub-optimal^e^
Discharged^f^	optimal^f^	optimal^f^	optimal^f^
Multiple outcomes			
Consultant & imaging^k^	sub-optimal^k^	sub-optimal^k^	sub-optimal^k^
Consultant & injection^k^	sub-optimal^k^	sub-optimal^k^	sub-optimal^k^
Consultant & physio^k^	sub-optimal^k^	sub-optimal^k^	sub-optimal^k^
Dietician & discharged^m^	sub-optimal^m^	sub-optimal^m^	sub-optimal^m^
Dietician & Imaging^i^	optimal^i^	optimal^i^	optimal^i^
Dietician & injection^i^	optimal^i^	optimal^i^	optimal^i^
Dietician & injection & discharged^i^	optimal^i^	optimal^i^	optimal^i^
Dietician & monitoring^g^	sub-optimal^g^	sub-optimal^g^	sub-optimal^g^
Discharged & other speciality^h^	optimal^h^	optimal^h^	optimal^h^
Imaging & discharged^n^	optimal^n^	optimal^n^	optimal^n^
Imaging & injection^i^	optimal^i^	optimal^i^	optimal^i^
Imaging & other speciality^g^	sub-optimal^g^	sub-optimal^g^	sub-optimal^g^
Injection & discharged^n^	optimal^n^	optimal^n^	optimal^n^
Injection & other speciality^g^	sub-optimal^g^	sub-optimal^g^	sub-optimal^g^
Monitoring & imaging^g^	sub-optimal^g^	sub-optimal^g^	sub-optimal^g^
Monitoring & imaging & injection^g^	sub-optimal^g^	sub-optimal^g^	sub-optimal^g^
Monitoring & injection^g^	sub-optimal^g^	sub-optimal^g^	sub-optimal^g^
Monitoring & other speciality^g^	sub-optimal^g^	sub-optimal^g^	sub-optimal^g^
Physio & dietician & discharged^n^	optimal^n^	optimal^n^	optimal^n^
Physio & dietician & injection^i^	optimal^i^	optimal^i^	optimal^i^
Physio & dietician & monitoring^g^	sub-optimal^g^	sub-optimal^g^	sub-optimal^g^
Physio & discharged^n^	optimal^n^	optimal^n^	optimal^n^
Physio & imaging^i^	optimal^i^	optimal^i^	optimal^i^
Physio & imaging & injection^i^	optimal^i^	optimal^i^	optimal^i^
Physio & injection^i^	optimal^i^	optimal^i^	optimal^i^
Physio & monitoring^g^	sub-optimal^g^	sub-optimal^g^	sub-optimal^g^
Physio & monitoring & imaging^g^	sub-optimal^g^	sub-optimal^g^	sub-optimal^g^
Physio & monitoring & injection^g^	sub-optimal^g^	sub-optimal^g^	sub-optimal^g^
Physio & monitoring & otherspeciality^g^	sub-optimal^g^	sub-optimal^g^	sub-optimal^g^
Physio & otherspeciality^l^	optimal^l^	optimal^l^	optimal^l^
Physio & surgery^l^	optimal^l^	optimal^l^	optimal^l^
Surgery & imaging^l^	optimal^l^	optimal^l^	optimal^l^

SINGLE OUTCOMES:

^a^Consultant outcome is sub-optimal for all clinic types because it introduces an additional referral, adding a superfluous step in the pathway

^b^Physio or Dietician are sub-optimal outcomes for orthopaedic clinic only because of backtracking to a specialist clinician in non-surgical treatments rather than referring to them directly from primary care

^c^Monitoring is sub-optimal for all clinic types as it creates additional consultation

^d^Surgery is sub-optimal for advanced physiotherapy practitioner and specialist GP as better resource use would be to refer to an orthopaedic clinic directly

^e^Imaging and injection were not routinely available to General Practitioner referrers. They were considered optimal outcomes in advanced physiotherapy practitioner or specialist GP, but not for orthopaedic clinic as surgeon time was not required for this

^f^Discharge is considered optimal as it is a definitive treatment

MULTIPLE OUTCOMES:

^g^Monitoring or OtherSpeciality in combination with other treatments (except discharge) were sub-optimal as they do not qualify as definitive treatment outcome

^h^Discharge and OtherSpeciality which was optimal for all clinic types as a definitive treatment was given

^i^Imaging and/ or Injection were not routinely available to General Practitioner referrers so as non-surgical treatments they were considered optimal in advanced physiotherapy practitioner or specialist GP clinic. Imaging and/ or injection were considered optimal for orthopaedic clinic if combined with other non-surgical treatments such as physio or dietician

^k^Consultant combined with multiple other outcomes were sub-optimal as there was no definitive outcome and introduce superfluous steps in the pathway

^l^Physio and Surgery or OtherSpeciality were optimal as there was a definitive treatment outcome

^m^Dietician and Discharge for all clinic types was sub-optimal outcome as there was specific local General Practitioner referral guidance around weight management

^n^Discharge in combination with other treatments (except dietician) is optimal as it is a definitive treatment outcome

All data were analysed using SPSS (version 20) and STATA (version 13). The HOOS and KOOS scores were combined as a single measure as the analysis was not specific to joint type and to manage the large volume of data based on the analysis of the individual sub scales for pain, symptoms, sports, function and quality of life. The score for each sub-scale was calculated by transforming each score to a 0–100 scale, with zero representing extreme knee problems and 100 representing no knee problems. A combined pain and function KOOS_2_/HOOS_2_ score was calculated using the method described by [23] to reduce the number of variables.

Baseline demographic and clinical data together with the text mining outputs from referral letters were summarised and tabulated. Frequencies were also calculated to describe important characteristics of the care pathway. A Chi square test was used to determine association between optimal pathway and clinic type, i.e. orthopaedic clinic, advanced physiotherapy practitioner or specialist GP. Logistic regression was used to investigate demographic, clinical and text concept variables associated with an optimal pathway. Variables associated at the 10% level in univariate analyses were included in a multivariate model. Variables in the multivariate model were entered using a stepwise backward selection process, with all candidate variables initially entered, and variables not significant at the 10% level sequentially removed one by one until the model could no longer be improved. A similar modelling strategy was employed for the investigation of factors predictive of KOOS/HOOS combined pain and function scores, in this case linear regression was used for continuous data. An independent T-test was used for the comparison of mean combined KOOS/HOOS score at 6 months between those on optimal and suboptimal care pathways. At 6 months post consultation predictors of combined pain and function scores were explored using a univariate analysis only.

## Results

**Fig. 2:**
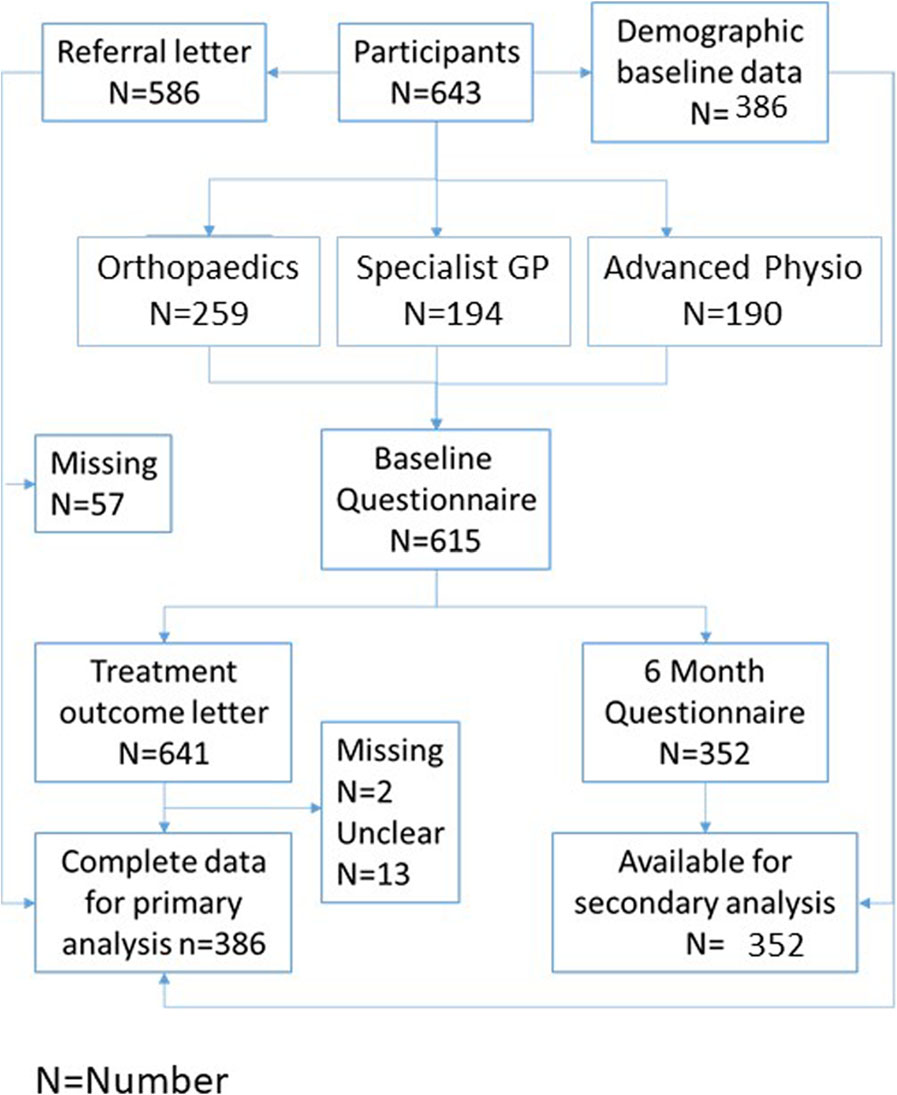
Study flow chart

A total of 643 participants were recruited. Referral letters were available for 586 out of 643 (91.1%) participants. The study flow chart in Fig. 2 provides details of the data included in the analysis. Based on the automatically extracted codes from the free text content of the referral letters, a total of 14 pertinent variables were identified from a set of 49 possible variables. The definition of each variable taken from the Unified Medical Langauge System [24] can be found Table 7 in Appendix.

### Factors predicting an optimal care pathway (primary aim)

Overall, 419/653 (66.7%) participants were classified as having an optimal pathway. Participants seen in orthopaedic clinic type were more likely to follow an optimal care pathway 192/255 (75.3%). This association between optimal/sub-optimal pathway and clinic type was statistically significant (*p* < 0.001) (see Table 2).

**Table 2:** Optimal care pathway by clinic type

		Optimal pathway						Chi Sq *p*-value
		Sub optimal (*n* = 209)		Optimal (*n* = 419)		Total (*n* = 628)		
		number	%	number	%	number	%	
Type of clinic	specialist GP	61	32.6	126	67.4	187	100.0	< 0.001
	Advanced physiotherapy practitioner	85	45.7	101	54.3	186	100.0	
	Orthopaedic clinic	63	24.7	192	75.3	255	100.0	

**Fig. 3:**
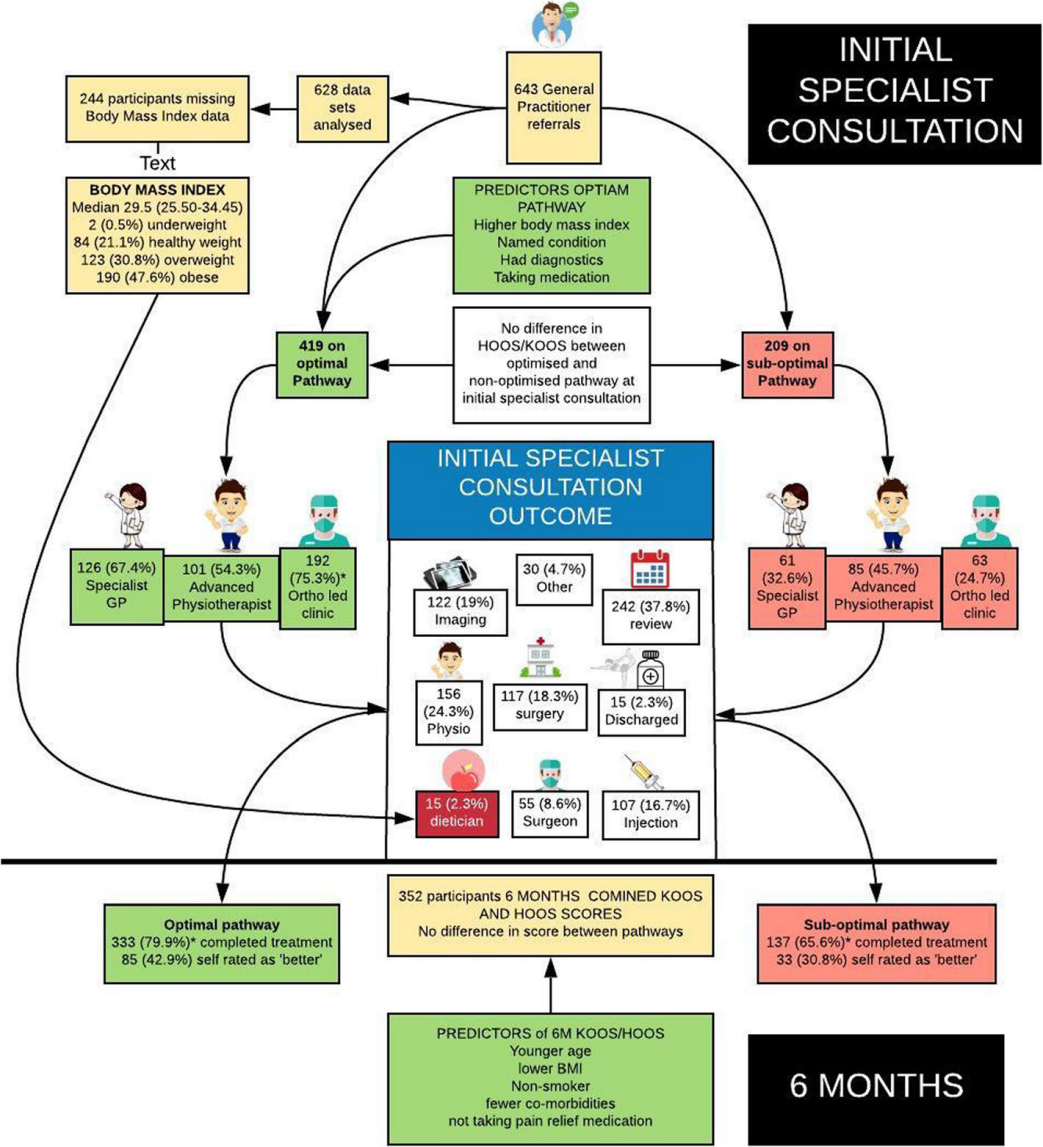
Treatment outcomes and optimal care from patients seen on the current hip and knee pathway

Factors in the final multivariate logistic regression analysis that predicted optimal care pathway (regardless of clinic type) were: lower BMI, having an explicitly named disease or syndrome and taking a pharmacologic substance. While having multiple diagnostic procedures was associated with an optimal pathway, this association was not statistically significant whereas having a single diagnostic procedure was significantly associated with a suboptimal pathway. Results of the multivariate regression are listed in Table 3. Figure 3 details the number of participants recruited, clinic type, consultation outcomes and predictor variables at initial consultation.

**Table 3:** Logistic regression analysis for optimal referral pathway-all participants

Variables		Sub optimal (*n* = 209)		Optimal (*n* = 419)		Final multivariate regression (*n* = 386)		
		number	%	number	%	Odds ratio	95% confidence interval	*p*-value
BMI score		136	32.3 (8.8)	257	30.0 (6.7)	1.0	0.9, 1.0	0.004
Diagnostic procedure	0	52	32.3	109	67.7	Ref		0.001
	1	107	37.3	177	62.3	0.5	0.3, 0.9	
	2+	33	25.6	96	74.4	1.4	0.7, 2.8	
Disease or syndrome	No	87	40.1	130	59.9	Ref		0.017
	Yes	105	29.4	252	70.6	1.8	1.1, 2.8	
Pharmacologic substance	No	163	35.3	299	64.7	Ref		0.039
	Yes	29	25.9	83	74.1	1.8	1.0, 3.3	

Variables not included in the final multivariate model as they were not significant at 10% level: age, gender, smoking status, welsh index of multiple deprivation (median and quintiles), geographical area, joint type, BMI categories, Charleston co-morbidity index (median and score) number of co-morbidities, medication history, number of semantic types, referral letter length, body related concept, daily or recreational activity, finding, functional concept, health and care activity, injury or poisoning, occupational activity, physiologic function, sign or symptom, tissue, therapeutic or preventative procedure

### Factors predicting baseline combined HOOS and KOOS scores for pain and function at initial consultation and after 6 months (secondary aim)

Factors that predicted patient rated combined KOOS and HOOS for pain and function across all participants at time of initial specialist consultation, regardless of clinic type or pathway are summarised in Table 4.

**Table 4:** Predictors of the combined baseline HOOS and KOOS score

			Combined KOOS and HOOS			Final linear regression (number = 319)	
			number	Univariate coefficient (95% confidence interval)	*p*-value	Multivariate coefficient (95% confidence Interval)	*p*-value*
Age at baseline			601	−0.4 (− 0.5, − 0.3)	< 0.001	−0.2 (− 3.5, − 0.1)	0.009
BMI score mean (standard deviation)			373	− 0.9 (− 1.2, − 0.6)	< 0.001	−0.75, (− 1.1, − 0.4)	< 0.001
Smoking data		Non-smoker	406	Ref	0.021	Ref	< 0.001
		Smoker		−8.7 (−15.3, −2.1)		−14.0 (− 20.5, − 7.5)	
		Former smoker		−4.7 (− 10.2, 0.8)		1.3 (− 4.0, 6.5)	
Joint type		Hip	601	Ref	0.003	Ref	0.010
		Knee		7.7 (2.7, 12.8)		7.9 (1.9, 13.9)	
Sign or symptom		No	549	Ref	0.002	Ref	0.030
		Yes		16.6 (6.2, 27.0)		12.0 (1.2, 22.8)	
Therapeutic or preventive procedure		No	549	Ref	< 0.001	Ref	0.003
		Yes		−7.9 (−11.9, −3.9)		−6.9 (−11.5, −2.3)	
Medication history	Non-opioid	No	429	Ref	< 0.001	Ref	
		Yes		−16.3 (−23.7, −9.0)		−10.6 (−17.6, −3.6)	0.003
	Opioids	No	429	Ref	< 0.001	Ref	< 0.001
		Yes		−18.3 (−23.0, −13.6)		−14.47 (−19.4, −9.5)	

Variables not included in the final multivariate model as they were not significant at 10% level: gender, Welsh index of multiple deprivation (median and quintiles), geographical area, joint type, BMI categories, Charleston co-morbidity index (median and score) number of co-morbidities, medication history, number of semantic types, referral letter length, body related concept, diagnostic procedure, daily or recreational activity, disease or syndrome, finding, functional concept, health and care activity, injury or poisoning, occupational activity, physiologic function, pharmacologic substance, tissue, therapeutic or preventative procedure.

*NSAID* Non-steroidal anti-inflammatory drug

*variables significantly associated with combined HOOS/KOOS score at 10% level

At 6 months after initial consultation there was no statistically significant difference in combined KOOS and HOOS scores for pain and function between the optimal (mean 62.6, SD 26.6) and sub-optimal pathway (mean 57.6, SD 22.2) (T-test, *p* = 0.112). Predictors of combined KOOS and HOOS scores at 6 months are summarised in Table 5. Figure 3 details the number of participants recruited, clinic type, consultation outcomes and predictor variables at 6 month’s follow-up.

**Table 5:** Predictors for 6 month KOOS, HOOS combined pain and function in daily living score

			number	Mean (standard deviation) combined KOOS and HOOS	*p*-value
Age at baseline			341		0.010*
Body mass index score			219		0.001*
Body mass index categories		Healthy weight	40	66.6 (29.4)	0.002*
		Overweight	70	61.5 (25.3)	
		Obese	109	52.0 (22.6)	
Smoking data		Non-smoker	131	63.9 (24.7)	0.011*
		Smoker	34	55.8 (27.4)	
		Former smoker	75	53.3 (25.1)	
Charlson comorbidity index			193		< 0.001*
Charlson score category		0	128	63.9 (23.4)	< 0.001*
		1	56	56.4 (23.5)	
		2	34	53.4 (28.1)	
		3+	15	37.3 (24.7)	
Medication history	Non-opioid	No	212	60.9 (24.7)	0.019*
		Yes	29	49.2 (27.3)	
	Non-steroidal anti-inflammatory drugs	No	171	61.3 (24.87)	0.097
		Yes	70	55.3 (25.7)	
	Opioids	No	173	64.0 (23.5)	< 0.001*
		Yes	68	48.1 (26.0)	
	Neuropathic agents	No	214	61.7 (24.4)	0.001*
		Yes	27	42.2 (24.9)	
Gender		Male	147	63.0 (24.9)	0.098
		Female	168	58.5 (24.9)	
Welsh index of multiple deprivation			341		0.093
Welsh index of multiple deprivation quintiles		1 to 382	93	54.8 (25.9)	0.066
		383 to 764	54	62.9 (24.9)	
		765 to 1146	82	65.0 (22.5)	
		1147 to 1528	22	58.6 (26.0)	
		1529 to 1909	90	62.5 (25.1)	
Geographical area		Vale of Glamorgan	149	59.1 (25.9)	0.255
		Cardiff	192	62.2 (24.2)	
Joint type		Hip	58	59.1 (25.5)	0.571
		Knee	283	61.2 (24.9)	

*variables significantly associated with combined HOOS/KOOS score at 10% level

*KOOS/HOOS* Knee/hip osteoarthritis outcome score

### Pathway characteristics by clinic type (secondary aim)

The characteristics of the participants per clinic type are listed in Table 6. A total of 535 participants had knee pain (83.2%) and 108 had hip pain (16.8%). All participants who had hip pain received their specialist consultation in orthopaedic clinic. Regardless of clinic types, the largest proportion of patients were non-smokers and had a BMI, that was categorised as obese. Fewer individuals in the advanced physiotherapy practitioner clinic were taking prescribed pain medication, they also had the lowest Charleston co-morbidity index and highest mean KOOS pain and function sub-scale scores. Descriptive data of the coded variables extracted from the referral letter per clinic type are listed in presented Table 8 in Appendix.

**Table 6:** Participant characteristics for demographic factors from referral letters, scores from combined KOOS/HOOS scores and clinic outcome per clinic type

		Type of clinic							
		Specialist GP		Advanced physiotherapy practitioner		Orthopaedic			
		n	% or mean (SD)	n	% or mean (SD)	n	% or mean (SD)	n	% or mean (SD)
Participant characteristics from referral letters									
Age at baseline mean (SD)		194	59.2 (13.9)	190	41.8 (13.6)	259	57.8 (16.9)	643	53.5 (16.9)
Gender	Male	96	49.5	92	48.4	123	47.5	311	48.4
	Female	98	50.5	98	51.6	136	52.5	332	51.6
Smoking data	Non-smoker	66	47.8	91	64.1	91	58.3	248	56.9
	Smoker	26	18.8	28	19.7	18	11.5	72	16.5
	Former smoker	46	33.3	23	16.2	47	30.1	116	26.6
Welsh index of multiple deprivation median (IQR)		194	825 (346, 1459)	190	825 (346, 1576)	259	825 (346, 1668)	643	825 (346, 1545.5)
WIMD quintiles	1 to 382	49	25.3	57	30.0	83	32.0	189	29.4
	383 to 764	44	22.7	29	15.3	40	15.4	113	17.6
	765 to 1146	44	22.7	39	20.5	52	20.1	135	21.0
	1147 to 1528	16	8.2	16	8.4	13	5.0	45	7.0
	1529 to 1909	41	21.1	49	25.8	71	27.4	161	25.0
BMI categories	BMI score Median (IQR)	115	30.1 (26.5, 35.0)	136	28.5 (24.6, 33.0)	148	30.5 (25.8, 35.3)	399	29.5 (25.5, 34.5)
	Underweight	0	0.0	0	0.0	2	1.4	2	0.5
	Healthy weight	14	12.2	41	30.1	29	19.6	84	21.1
	Overweight	41	35.7	43	31.6	39	26.4	123	30.8
	Obese	60	52.2	52	38.2	78	52.7	190	47.6
Medication history	No medication	17	8.8	21	11.1	8	3.1	46	7.2
	With medication^a^	129	66.5	133	70.0	151	58.3	413	64.2
	Non-opioid	16	11.0	9	5.8	22	13.8	47	10.2
	NSAIDS	36	24.7	31	20.1	51	32.1	118	25.7
	Opioids	44	30.1	29	18.8	58	36.5	131	28.5
	Neuropathic agents	21	14.4	9	5.8	21	13.2	51	11.1
Charleston co-morbidity index	Index† Median (IQR)		0.0 (0.0, 1.0)		0.0 (0.0, 0.0)		0.0 (0.0, 2.0)		0.0 (0.0, 1.0)
	0	81	58.3	98	67.6	77	52.0	256	59.3
	1	37	26.6	32	22.1	31	20.9	100	23.1
	2	13	9.4	10	6.9	22	14.9	45	10.4
	3+	8	5.8	5	3.4	18	12.2	31	7.2
Combined KOOS/HOOS scores									
Combined KOOS/ HOOS sub-scale scores	Pain (mean (SD))	187	48.1 (20.1)	179	58.0 (22.4)	236	45.7 (22.4)	602	50.1 (22.3)
	Other symptoms (mean (SD))	189	51.7 (19.9)	184	56.7 (20.2)	239	48.4 (21.8)	612	51.9 (21.0)
	Function in daily living (mean (SD))	187	53.0 (23.4)	182	63.8 (24.3)	237	49.3 (25.1)	606	54.8 (25.1)
	Sport and recreation function (median (IQR))	154	25.0 (10.0, 50.0)	172	35.0 (20.0, 62.5)	207	25.0 (5.0, 50.0)	533	30.0 (10.0, 55.0)
	Quality of life (median (IQR))	184	31.3 (12.5, 43.8)	181	37.5 (18.8, 50.0)	234	25.0 (12.5, 43.8)	599	31.3 (12.5, 43.8)
Consultation outcome									
Number of treatment outcome types received	0	6	3.1	3	1.6	4	1.5	13	2.0
	1	65	33.7	71	37.6	182	70.3	318	49.6
	2	98	50.8	85	45.0	58	22.4	241	37.6
	3	22	11.4	27	14.3	12	4.6	61	9.5
	4	2	1.0	3	1.6	3	1.2	8	1.2
Number of treatments for each treatment outcome type	Consultant	16	8.3	29	15.3	10	3.9	55	8.6
	Physio	38	19.7	102	54.0	16	6.2	156	24.3
	Dietician	9	4.7	4	2.1	2	0.8	15	2.3
	Review appointment	64	33.2	97	51.3	81	31.3	242	37.8
	Surgery	0	0.0	1	0.5	116	44.8	117	18.3
	Imagining	23	11.9	45	23.8	54	20.8	122	19.0
	Injection	78	40.4	5	2.6	24	9.3	107	16.7
	Discharged	104	53.9	42	22.2	38	14.7	184	28.7
	Podiatry or national exercise referral scheme	3	1.6	9	4.8	5	1.9	17	2.7
	Unknown	6	3.1	3	1.6	4	1.5	13	2.0

*n* number, *SD* standard deviation, *IQR* Interquartile range

^a^Definition of with medication = at least one (any) medication recoded in the medication data

#### Consultation outcomes per clinic type

Across all clinic types, a total of 53 different treatment outcome combinations were identified. Half of the participants had one treatment outcome, 306 (48.3%) had two or more treatment outcomes. The number and types of treatment outcomes identified from the outcome letters are detailed in Table 6.

## Discussion

The primary aim of this study was to identify factors from GP referral letters that can predict which patients with knee and/or hip pain would receive an optimal care pathway at the time of consultation. Factors which were found to predict an optimal care pathway were: lower BMI,, having a named disease or syndrome and taking a pharmacologic substance. Having a single diagnostic procedure predicted a sub-optimal pathway. Over 30% of participants were found not to have had an optimal care pathway. The secondary aims were to identify predictors of patient rated pain and function at time of consultation and after 6 months and to describe the characteristics of the care pathway. Variables found to predict pain and function at initial consultation were higher age, higher BMI, current smoking, with knee pain, having sign or symptoms and having therapeutic/preventative procedure and opioid medication history. Of these variables only age, BMI, smoking status and medication history were individually found to predict pain and function at 6 months post consultation. Only predictors related to BMI were predictors of both optimal care and pain and function. A key characteristic of the care pathway for individuals with knee and/ or hip pain is that treatments received varied according to the type of specialist clinic seen in.

### Predictors of receiving care on an optimal versus sub-optimal pathway at the time of initial consultation

Our findings suggest that not all patients received a treatment outcome that resulted in an optimal care pathway. This represents potential inefficiency and wasted healthcare resource use. In this particular cohort of patients this could be improved for over 30% of cases, which has not previously been quantified in the musculoskeletal literature. Variables associated with optimal care and predicted 10% of variance were lower BMI and three concepts from the free text of the referral: having a named disease or syndrome and taking a pharmacologic substance. Having a single diagnostic procedure predicted a sub-optimal care pathway. These factors should be routinely included in referrals as part of a minimum dataset. Despite BMI being a strong predictor it was frequently unreported, so addressing this in future referral guidelines is essential. Pain and function at time of consultation or at 6 months post consultation did not predict receiving optimal care. One explanation for this is that the definition of ‘an optimal pathway’ used in this study is about efficient resource allocation and does not consider patient rated pain and function. Therefore, efficient use of healthcare resources is not necessarily related to patient opinion of their condition and these are independent concepts.

### Predictors of patient rated pain and function at time of consultation

Factors that predicted baseline patient rated KOOS and HOOS for combined pain and function across all participants regardless of clinic type or pathway were higher age, higher BMI, current smoking, with knee pain, having sign and symptoms, having therapeutic/preventative procedure and opioid medication history. Individually, many of these variables were also found to predict combined KOOS and HOOS scores at 6 months: age, BMI, smoking status, co-morbidity index and medication history. This should be interpreted with caution as this is not part of the multivariate analysis but all of these factors should be routinely documented in referrals for specialist opinion.

Based on the study findings it is apparent that factors used to predict optimal care are different to those that predict pain and function. Furthermore, BMI was a predictor for both receiving optimal care and pain and function outcome, it is therefore essential that this is included in any future minimal dataset.

The methods using in this study are novel for triaging referrals for specialist opinion. No previous studies have evaluated care factors that predict who received optimal care, but these factors do need to be considered in any future referral or triage system. When developing prioritization tools for patient triage it is essential to include demographic data and variables that we have identified from the free text component of the referral.

Previous studies have evaluated triage prioritisation tools for hip and knee pain, but these were not based on predictor variables [10] and there had been a lack of transparency about what variable prioritisation is based on [11, 25, 26]. Further research is required to validate the findings of this study and to develop the prioritisation tools and training required for an optimal pathway that could be tested in a randomized control trial in the future. This staged approach is in line with the IDEAL-Physio framework for guiding innovation and evidencing interventions [27].

### Care pathway characteristics

A secondary aim of this study was to describe the characteristics of the care pathway for hip and / or knee pain at the point of referral for specialist assessment according to specialist clinic type and receiving care on an optimal or sub-optimal pathway. The care pathway that patients in this study followed is displayed in Fig. 3. The organisation of care around three different professional specialities (specialist GP, advanced physiotherapy practitioner and orthopaedics) represents further variation compared to that already described in the literature. For example, combined physiotherapist and orthopaedic clinics [28–32], separate orthopaedic and physiotherapist clinics [25, 33] or musculoskeletal clinical assessment triage and treatment service (MCATS) combing advanced physiotherapy practitioner and orthopaedic consultant, physiotherapy led clinics [34] separate Orthopaedic clinics [7].

In the current study the treatment outcomes are reported according to the clinic type. There was higher referral rate to conservative treatments and imaging by advanced physiotherapy practitioner, higher injection rate by specialist GP and higher rates of surgical intervention for participants seen in orthopaedic clinic. There is a scarcity of evidence in the literature around treatment outcomes for the different care pathways for hip and knee pain. Data that are available also suggest that patients seen in a physiotherapist or musculoskeletal care, assessment and treatment clinic are more likely to receive an injection, non-steroidal anti-inflammatory drug prescription, a course of physiotherapy or conservative treatment [6, 7, 32]. Therefore, there is a risk that there will be variation in treatment offered based on the professional background of the healthcare professional [7]. Furthermore, it could be argued that these patients should receive conservative treatments in primary care, before being referred for specialist opinion and represents inappropriate referral [1].

In addition, individuals seen in the advanced physiotherapy practitioner clinics tended to be younger, have lower BMI, have fewer co-morbidities, take fewer medication, have a higher level of function and less pain. This would seem to corroborate the finding that this group of individuals are less likely to require surgery and have a higher rate of conservative treatment options [7, 29].

The referral rates from advanced physiotherapy practitioner clinic for surgery/ surgical opinion are comparable to the literature, although high variation is reported, ranging from 9 to 66% [31, 33, 35]. A reason for this variation is the difference in clinic structure, i.e. multi-profession versus single profession clinics. Referral rates for MRI were similar to those reported in previous studies (13–23% referral rate) [7, 29, 33]. We found evidence that some treatments such as dietetics was underutilised, with a very low referral rate across clinic type despite high levels of patients that were classified as overweight or obese. Similar finding has been reported previously by [6, 36].

In the current study, participants were more likely to follow an optimal care pathway if seen in orthopaedic clinic and less likely if seen in advanced physiotherapy practitioner clinic. One reason for this is that patients seen in orthopaedic more frequently had one definitive treatment at the conclusion of the specialist consultation, whereas in advanced physiotherapy practitioner clinics patients more frequently had multiple treatment outcomes. Furthermore, higher numbers of patients were given review appointments for advanced physiotherapy practitioner clinics and this may be as a result of patients trying a range of conservative treatments and therefore the outcome of these was being monitored [20]. In the future additional methods of optimising the pathway could include adopting a combined skill mix of professions, providing training for primary care clinicians and developing methods for streamlining specialist referrals to the appropriate profession [5].

### Study limitations

There were missing values, especially around the BMI. Further limitations are concerned with the generalisability of findings as (1) there was a lower proportion of patients with hip pain and (2) data were collected from a single Health Board. The definition of ‘optimal pathway’ used in this study was based around efficient healthcare resource allocation in line with published guidelines and local policy/ referral guidance. The context of this study means that the application of ‘optimal and sub optimal’ pathway is subjective and will apply differently across different services. This does reflect the complexity and activity loops present within the care pathway [5]. This definition is limited as it does not take into consideration changes in the patient’s condition, patient opinion of their symptoms or characteristics and preferences of the referrers. There was inconsistency for the diagnostic procedure variable at predicting optimal care pathway. Two or more variables was associated with an optimal care pathway but one diagnostic procedure was associated with a sub-optimal care pathway. Therefore this variable needs to be interpreted with caution. Finally, it has not been established how many of those that were referred to a consultant ended up having surgery, which may have affected what was recorded as a treatment outcome, i.e. referral or surgery. Due to missing data a multivariate analysis was not conducted on KOOS/HOOS combined scores at 6 months post consultation.

## Conclusions

In this study 30% of individuals did not follow an optimal care pathway which represents potential inefficiency and wasted healthcare resource. A core set of variables from the free text of referrals has been identified that should be included in a minimum information standard when referring an individual for specialist opinion for knee and hip pain. Of key importance is data on BMI as this was a predictor for both optimal care and pain and function outcomes. Patient rated outcomes for pain and function on their own were not predictors of optimal care and therefore cannot be used on their own to streamline patient referrals. A high number of patients seen in the specialist GP and advanced physiotherapy practitioner clinics received conservative treatments that could have been carried out in primary care. Finally, there was variation in the type of treatment a patient received depending on the clinic type. The recommendation from this study would be to utilize the different skill-mix of the healthcare professionals in the pathway to improve referral to conservative treatments in primary care. Further validation of a core data set at predicting optimal care to streamline referrals is required.

## Data Availability

The datasets for the current study are available on request from the corresponding author on reasonable request.

## Appendix

**Table 7:** Unified medical language system semantic types

Description	Definition
Diagnostic procedure	A procedure, method, or technique used to determine the nature or identity of a disease or disorder. This excludes procedures which are primarily carried out on specimens in a laboratory.
Daily or recreational activity	An activity carried out for recreation or exercise, or as part of daily life.
Disease or syndrome	A condition which alters or interferes with a normal process, state, or activity of an organism. It is usually characterized by the abnormal functioning of one or more of the host's systems, parts, or organs. Included here is a complex of symptoms descriptive of a disorder.
Finding	That which is discovered by direct observation or measurement of an organism attribute or condition, including the clinical history of the patient. The history of the presence of a disease is a ‘Finding’ and is distinguished from the disease itself.
Functional concept	A concept which is of interest because it pertains to the carrying out of a process or activity.
Health care activity	An activity of or relating to the practice of medicine or involving the care of patients.
Injury or poisoning	A traumatic wound, injury, or poisoning caused by an external agent or force.
Occupational activity	An activity carried out as part of an occupation or job.
Physiologic function	A normal process, activity, or state of the body.
Pharmacologic substance	A substance used in the treatment or prevention of pathologic disorders. This includes substances that occur naturally in the body and are administered therapeutically.
Sign or symptom	An observable manifestation of a disease or condition based on clinical judgment, or a manifestation of a disease or condition which is experienced by the patient and reported as a subjective observation.
Tissue	An aggregation of similarly specialized cells and the associated intercellular substance. Tissues are relatively non-localized in comparison to body parts, organs or organ components.
Therapeutic or preventive procedure	A procedure, method, or technique designed to prevent a disease or a disorder, or to improve physical function, or used in the process of treating a disease or injury.
Body related concept	This is a custom concept and is a union of 3 concepts: Body Location or Region, Body Part, Organ or Organ Component and Body Space or Junction, whose definitions are provided below.
Body location or region	An area, subdivision, or region of the body demarcated for the purpose of topographical description.
Body part, organ, or organ component	A collection of cells and tissues which are localized to a specific area or combine and carry out one or more specialized functions of an organism. This ranges from gross structures to small components of complex organs. These structures are relatively localized in comparison to tissues.
Body space or junction	An area enclosed or surrounded by body parts or organs or the place where two anatomical structures meet or connect.

**Table 8:** Coded variables extracted from referral letters by clinic type

	Type of clinic							
	specialist GP		Advanced physiotherapy practitioner		Orthopaedic		Total	
	number	%	number	%	number	%	number	%
Body related concept	126	66.7	141	76.6	176	82.6	443	75.6
Diagnostic procedure	139	73.5	127	69.0	156	73.2	422	72.0
Daily or recreational activity	71	37.6	62	33.7	67	31.5	200	34.1
Disease or syndrome	130	68.8	111	60.3	125	58.7	366	62.5
Finding	116	61.4	113	61.4	147	69.0	376	64.2
Functional concept	33	17.5	35	19.0	52	24.4	120	20.5
Health care activity	68	36.0	56	30.4	98	46.0	222	37.9
Injury or poisoning	43	22.8	63	34.2	44	20.7	150	25.6
Occupational activity	29	15.3	37	20.1	46	21.6	112	19.1
Physiologic function	24	12.7	34	18.5	15	7.0	73	12.5
Pharmacologic or substance	39	20.6	21	11.4	55	25.8	115	19.6
Sign or symptom	182	96.3	181	98.4	203	95.3	566	96.6
Tissue	19	10.1	19	10.3	18	805	56	9.6
Therapeutic or preventive procedure	76	40.2	42	22.8	111	52.1	229	39.1

## Abbreviations

BMI: Body mass index
KOOS or HOOS: Knee or Hip osteoarthritis outcome score
Specialist GP: GP with specialist interest in musculoskeletal disorders
